# Concurrent emergency head and cervical spine CT utilization: temporal trends, diagnostic yield, and clinical implications

**DOI:** 10.1007/s10140-026-02538-3

**Published:** 2026-09-05

**Authors:** Eero Vartiainen, Teemu Luoto, Otso Arponen, Irina Rinta-Kiikka, Jussi Hirvonen

**Affiliations:** 1 Department of Radiology, Faculty of Medicine and Health Technology, Tampere University, Tampere University Hospital, Tampere, Finland; 2 Department of Neurosurgery, Faculty of Medicine and Health Technology, Tampere University, Tampere University Hospital, Tampere, Finland

**Keywords:** Computed tomography, Cervical spine fracture, Head injury, Diagnostic yield, Overutilization, Emergency department

## Abstract

**Purpose:**

To evaluate the diagnostic yield, temporal trends, and clinical implications of concurrent non-contrast head and cervical spine computed tomography (CT) in emergency department trauma patients over a 10-year period.

**Methods:**

This single-center retrospective cohort study included 9,359 consecutive patients who underwent concurrent head and cervical-spine CT for suspected trauma between May 2012 and April 2022. Patient demographics, trauma mechanisms, alcohol documentation, imaging findings, and management of cervical fractures were analyzed. Diagnostic yields were calculated overall and by age, mechanism (with focus on ground-level falls), and time period. Temporal trends and predictors of cervical-spine fracture were assessed using regression models.

**Results:**

Acute cervical-spine fractures were present in 4.9% (457/9,359) of patients, while intracranial or craniofacial abnormalities occurred in 23.9% (2,240/9,359). Concurrent abnormalities on both scans were rare (1.3%), with no significant overall association between positive head CT and cervical-spine fracture (OR 1.16, *p* = 0.174). Imaging volume increased five-fold over the decade, while diagnostic yields declined significantly for both cervical fractures (from 7.1% to 3.9%) and cranial abnormalities (from 28.5% to 22.0%; both *p* < 0.001). This decline was driven by a rising proportion of ground-level falls (from 47% to 61%), a low-energy mechanism with low yield. Of 457 cervical fractures, 19.9% were treated surgically.

**Conclusions:**

Despite a five-fold increase in concurrent head and cervical spine CT utilization, diagnostic yields declined markedly over the 10-year period, primarily driven by the growing dominance of low-energy ground-level falls. These findings highlight significant overutilization of routine concurrent imaging.

**Supplementary Information:**

The online version contains supplementary material available at https://doi.org/10.1007/s10140-026-02538-3.

## Introduction

Utilization of computed tomography (CT) for head and cervical spine imaging in trauma evaluation has increased markedly in emergency departments over the past two decades. This growth has been driven largely by expanded use of CT rather than plain radiographs, even in many low-risk presentations [1–3]. Overuse of head CT scans, particularly for minor head injuries, is largely driven by fear of malpractice litigation, defensive medicine, and patient or clinician anxiety. Other factors include pressure to improve emergency department efficiency, lack of strict adherence to clinical decision rules, and overestimation of risks by healthcare providers. In general, head injuries are associated with cervical spine injuries, and emergency assessment of head injury patients should include at least clinical cervical spine clearance [4]. In many institutions, head and cervical spine imaging is routinely ordered together in emergency settings. This approach is common, especially in older patients or those with altered mental status, as reliable clinical assessment can be challenging [5, 6]. Despite its utility in individual patients, unnecessary concomitant head and cervical spine imaging with CT also contributes to rising radiation exposure, increasing healthcare burden and costs, and emergency department crowding [7].

**Fig. 1:**
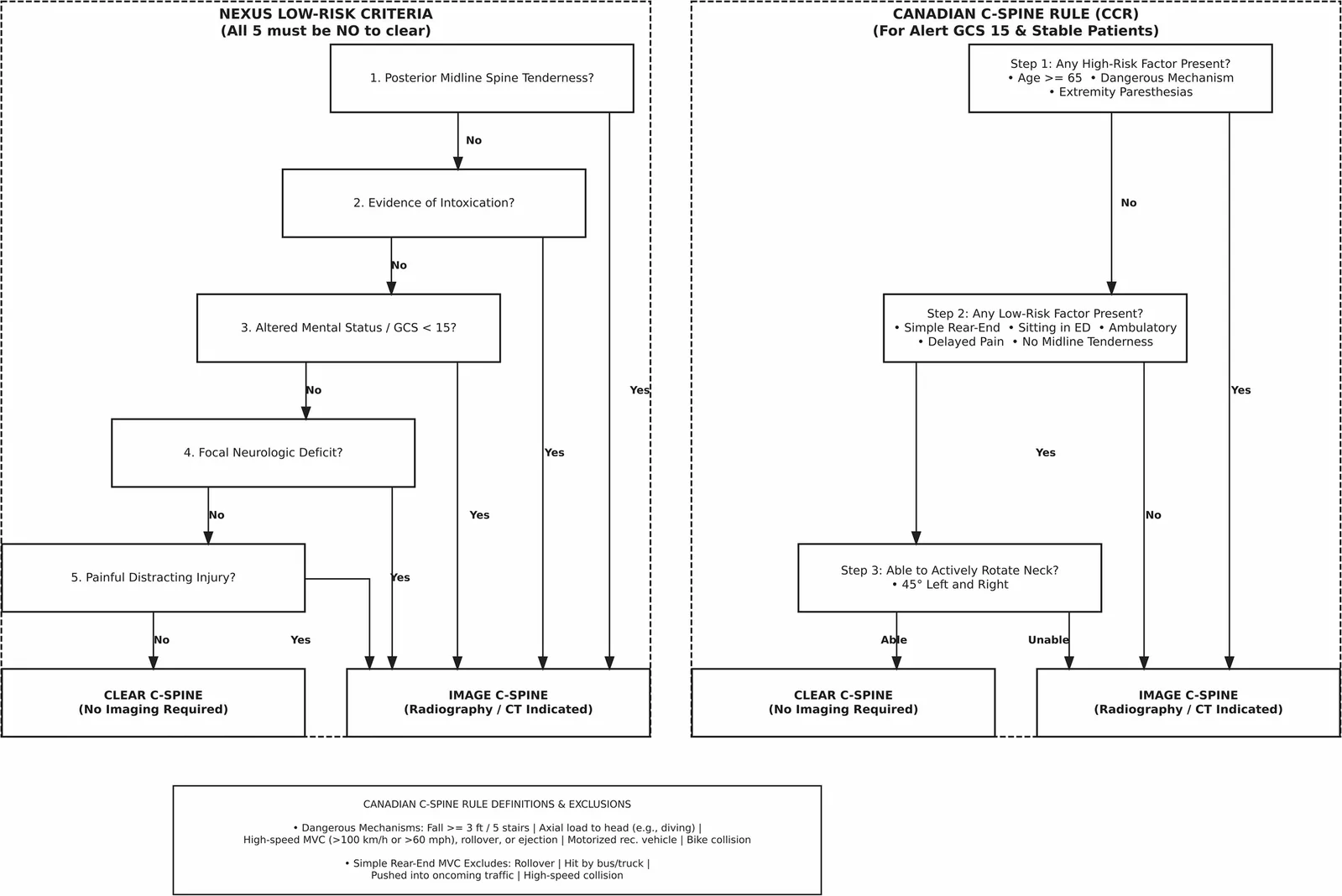
Summary of the National Emergency X-Radiography Utilization Study (NEXUS) criteria and the Canadian C-Spine Rule for selective cervical-spine imaging in blunt trauma

Published series report highly variable diagnostic yields: cervical spine fractures are found in 2–8% of scans, intracranial abnormalities in 15–35%, and simultaneous injury on both examinations in only 1–4% [8–10]. Several studies have documented a progressive decline in yield over time, attributed to lower imaging thresholds and an increasing proportion of low-energy mechanisms being used to justify referral, such as ground-level falls. Validated clinical decision rules (NEXUS [11], Canadian C-Spine Rule [12]; summarized in Fig. 1) can safely reduce cervical imaging by 25–50% in low-risk patients, yet adherence remains incomplete [13]. Most previous work has been limited by small sample sizes, short study periods, or selected cohorts, leaving uncertainty regarding real-world temporal trends and the contribution of specific mechanisms in large consecutive populations.

We therefore analyzed 9,359 consecutive concurrent head and cervical spine CT examinations performed over a full decade at a high-volume tertiary emergency department. The objectives were to determine age-stratified prevalence and co-occurrence of acute traumatic abnormalities, quantify changes in imaging volume and diagnostic yield over time, and examine the potential role of ground-level falls as a driver of increasing utilization and declining yield.

## Materials and methods

This single-center retrospective cohort study was conducted at a high-volume tertiary care hospital in Finland. The hospital serves as the only emergency department for a primary catchment population of approximately 530,000 inhabitants and acts as the regional Level 1 trauma center for a wider collaborative area with a population of 893,000. The department provides both primary and tertiary emergency care and manages 90,000–100,000 visits annually. The study was approved by the institutional review board. According to Finnish legislation, ethics committee approval and written informed consent were not required for this retrospective registry-based study.

All non-contrast head and cervical spine CT examinations performed in the emergency department between May 1, 2012, and April 30, 2022, were identified through the radiology information system. Inclusion required both a dedicated head CT and a dedicated cervical-spine CT acquired during the same emergency department visit for suspected trauma. Examinations performed as part of a whole-body trauma protocol were excluded, as were cases with missing or incomplete radiological reports. For patients with multiple examinations, only the first imaging episode was included. This yielded a final consecutive cohort of 9,359 patients who underwent concurrent non-contrast head and cervical-spine CT during the 10-year study period.

Patient demographics (age and sex) were retrieved from the electronic health records using the unique Finnish personal identity number. Three age strata were used: pediatric (0–17 years), working-age adults (18–64 years), and older patients (≥ 65 years). Clinical referral texts for all included examinations were individually reviewed by the first author. The primary trauma mechanism was recorded; when multiple mechanisms were mentioned, the highest-energy or most likely causative mechanism was selected. Explicit mention of alcohol intoxication in the referral text was also recorded.

Radiological findings were extracted from the original clinical reports. In accordance with institutional protocol, all after-hours head and neck CT examinations are routinely re-evaluated the following workday by a subspecialized neuroradiologist (unless the primary report was already issued by a neuroradiologist). Both the primary and secondary reports were reviewed, and acute traumatic abnormalities and incidental findings were recorded. The following lesions were recorded according to the NINDS common data elements (CDE) [14], based on a previous article [15]: Acute traumatic intracranial findings included subdural (SDH), epidural (EDH), intraparenchymal hemorrhage (intracerebral hematoma and hemorrhagic contusion), traumatic subarachnoid hemorrhage (SAH), midline shift, pneumocephalus, and diffuse axonal injury. In addition, we included hydrocephalus. Diffuse axonal injury cannot be definitively diagnosed on CT and was therefore recorded as a presumptive finding based on characteristic microhemorrhages and typical locations. Craniofacial findings comprised skull and facial bone fractures. Cervical spine fractures were classified by vertebral level (C0–C7); odontoid fractures were distinguished from other C2 fractures, and fractures were further categorized as upper cervical (C0–C2) or subaxial (C3–C7).

For all patients with at least one acute cervical spine fracture, the electronic medical records were reviewed to determine whether operative stabilization or conservative management was chosen during the index admission or subsequent follow-up, and whether follow-up imaging (CT, MRI, or radiography) was performed.

### Imaging technique

All examinations were performed on multi-detector CT scanners. From 2017 onward, the majority were acquired using a dual-source scanner (Somatom Definition Flash; Siemens Healthineers, Forchheim, Germany). In earlier years, a 64-row scanner (LightSpeed VCT; GE Healthcare, Waukesha, Wisconsin, USA) was used. Non-contrast head CT was acquired from the vertex to the upper cervical spine at 120 kV with automated tube current modulation and 0.5–1.0 mm slice thickness, with multiplanar reformats. Cervical spine CT was performed from the occiput to T4 using the same settings and 0.6–1.0 mm slice thickness, with routine sagittal and coronal bone reformats. Iterative reconstruction was applied when available.

### Statistical analysis

Continuous variables (e.g., age) are expressed as mean ± standard deviation and median (range). Categorical variables are presented as counts and percentages. Diagnostic yield was calculated as the proportion of examinations with at least one acute traumatic finding.

Differences in continuous variables between groups were assessed using the Mann–Whitney U test. Time trends in annual imaging volume and diagnostic yield were evaluated by comparing the periods 2012–2017 and 2017–2022 using the non-parametric Mann–Whitney U test, and linear regression was used to estimate annual changes in yield. Associations between categorical variables (e.g., acute cranial and cervical spine injury, documented alcohol intoxication, and imaging findings) were analyzed using the χ² test; odds ratios with 95% confidence intervals were calculated where relevant. Multivariable logistic regression models were developed to identify independent predictors of acute cervical spine fracture and any traumatic finding (head or spine). These models included age group, patient gender, and trauma mechanism as independent variables to calculate adjusted odds ratios (aOR) with 95% confidence intervals. To account for the five-fold increase in annual imaging volume, annual trends in diagnostic yield were evaluated using linear regression models weighted by annual scan volume.

Statistical analyses were performed using Microsoft Excel (Microsoft Corp., Redmond, WA, USA) and R statistical software (R Foundation for Statistical Computing, Vienna, Austria). Figures were also generated using R. Two-sided p-values < 0.05 were considered statistically significant.

## Results

A total of 9,359 patients underwent concurrent non-contrast head and cervical-spine CT examinations in the emergency department for suspected traumatic injury between May 1, 2012, and April 30, 2022. Patient demographics and referred trauma mechanisms are summarized in Table 1. The mean age of the cohort was 60.5 ± 24.1 years (median 66 years, range 0–106 years), with patients aged ≥ 65 years forming the largest group (*n* = 4,835, 51.7%), followed by adults aged 18–64 years (*n* = 4,117, 44.0%) and pediatric patients aged 0–17 years (*n* = 407, 4.4%). Males accounted for 51.8% (4,844/9,359; 95% CI 50.7–52.8%, *p* < 0.001) of the cohort.

**Table 1:** Patient demographics

	All cases	0–17	18–64	65+
Number of cases	9,359	407 (4.3%)	4,117 (44.0%)	4,835 (51.7%)
Age, mean; SD	60.5 (24.1)	13.0 (4.1)	42.0 (14.3)	80.3 (8.4)
Age, median; range	66 (0–106)	15 (0–17)	43 (18–64)	81 (65–106)
Sex				
Male	4,844 (51.8%)	217 (2.3%)	2,516 (26.9%)	2,111 (22.6%)
Female	4,515 (48.2%)	190 (2.0%)	1,601 (17.1%)	2,724 (29.1%)
Trauma mechanism				
Ground-level fall	5,313 (56.8%)	38 (9.3%)	1,448 (35.2%)	3,827 (79.2%)
Falling from height	768 (8.2%)	57 (14.0%)	410 (10.0%)	301 (6.2%)
Falling, unspecified	6 (0.1%)	0 (0.0%)	2 (0.0%)	4 (0.1%)
Motor vehicle accident (driver/passenger)	503 (5.4%)	38 (9.3%)	394 (9.6%)	71 (1.5%)
Violence	526 (5.6%)	12 (2.9%)	490 (11.9%)	24 (0.5%)
Bicycle accident	425 (4.5%)	43 (10.6%)	313 (7.6%)	69 (1.4%)
Traffic accident, pedestrian	52 (0.6%)	8 (2.0%)	30 (0.7%)	14 (0.3%)
Motorcycle/moped/snowmobile/quadbike/other motor vehicle accident	147 (1.6%)	68 (16.7%)	71 (1.7%)	8 (0.2%)
Electric scooter accident	93 (1.0%)	3 (0.7%)	89 (2.2%)	1 (0.0%)
Equestrian related accident	194 (2.1%)	41 (10.1%)	150 (3.6%)	3 (0.1%)
Sports related accident	222 (2.4%)	71 (17.4%)	140 (3.4%)	11 (0.2%)
No trauma referred	229 (2.4%)	1 (0.2%)	93 (2.3%)	135 (2.8%)
Other	274 (2.9%)	21 (5.2%)	210 (5.1%)	43 (0.9%)
Unknown/unspecified	607 (6.5%)	6 (1.5%)	277 (6.7%)	324 (6.7%)

Mean patient age increased steadily over the study period, by approximately 1.4 years per calendar year (from 52.6 years in 2012 to 67.5 years in 2022; *p* < 0.001), and the proportion of patients aged ≥ 65 years nearly doubled, from 36% to 66%. Ground-level fall was the dominant mechanism (*n* = 5,313, 56.8%), particularly among older patients (79.2%), whereas higher-energy mechanisms predominated in younger patients (Table 1).

### Cervical spine findings

Overall, acute cervical spine fractures were present in 457 of the 9,359 patients (4.9%). The incidence increased markedly with age: 7/407 (1.7%) in children, 133/4,117 (3.2%) in adults aged 18–64 years, and 317/4,835 (6.6%) in patients aged ≥ 65 years (*p* < 0.001).

A total of 669 individual fractures were recorded (mean 1.46 per injured patient); multilevel fractures occurred in 170 patients (37% of those with any fracture) and were especially frequent in the older patients (129/317, 41%). The distribution by vertebral level is shown in Table 2. C2 was the most commonly fractured vertebra (*n* = 202, 30% of all fractures), of which 118 (59%) were odontoid fractures. Upper cervical fractures (C0–C2) accounted for 79% of fractures in patients ≥ 65 years, whereas subaxial fractures were relatively more common in younger adults. Pediatric cervical spine fractures were rare and showed no clear level predominance. Incidental spinal tumors were detected in 16 patients (0.2%), almost exclusively in the older patients.

**Table 2:** Cervical spine fractures

	Total	0–17	18–64	65+
C0	24 (3.6%)	0 (0.0%)	11 (5.9%)	13 (2.7%)
C1	83 (12.4%)	0 (0.0%)	12 (6.5%)	71 (15.0%)
C2 (odontoid)	118 (17.6%)	3 (33.3%)	14 (7.5%)	101 (21.3%)
C2 (non-odontoid)	84 (12.6%)	0 (0.0%)	25 (13.4%)	59 (12.4%)
C3	42 (6.3%)	0 (0.0%)	11 (5.9%)	31 (6.5%)
C4	48 (7.2%)	0 (0.0%)	17 (9.1%)	31 (6.5%)
C5	82 (12.3%)	2 (22.2%)	21 (11.3%)	59 (12.4%)
C6	104 (15.5%)	2 (22.2%)	36 (19.4%)	66 (13.9%)
C7	84 (12.6%)	2 (22.2%)	39 (21.0%)	43 (9.1%)

Percentage of all fractures (C0-C7)

### Intracranial and craniofacial findings

At least one acute traumatic intracranial or craniofacial abnormality was identified in 2,240 of the cases (23.9%). The incidence increased significantly with age: 55/407 (13.5%) in children, 940/4,117 (22.8%) in adults aged 18–64 years, and 1,245/4,835 (25.7%) in patients aged ≥ 65 years (*p* < 0.001). The most frequent abnormalities were subdural hematoma (1,089 patients, 11.6%), intraparenchymal hemorrhage (694, 7.4%), facial fracture (677, 7.2%), traumatic subarachnoid hemorrhage (662, 7.1%), and skull fracture (458, 4.9%). All other findings occurred in ≤ 3.2% of the cohort (Table 3).

**Table 3:** Craniofacial abnormalities

	Total	0–17	18–64	65+
SDH	1,089 (24.0%)	14 (15.1%)	401 (20.4%)	674 (27.2%)
Intraparenchymal hemorrhage	694 (15.3%)	14 (15.1%)	292 (14.9%)	388 (15.6%)
EDH	77 (1.7%)	9 (9.7%)	52 (2.6%)	16 (0.6%)
SAH	662 (14.6%)	4 (4.3%)	269 (13.7%)	389 (15.7%)
Midline shift	302 (6.7%)	1 (1.1%)	103 (5.2%)	198 (8.0%)
Skull fracture	458 (10.1%)	20 (21.5%)	254 (12.9%)	184 (7.4%)
Facial fracture	677 (14.9%)	24 (25.8%)	373 (19.0%)	280 (11.3%)
Pneumocephalus	127 (2.8%)	2 (2.2%)	79 (4.0%)	46 (1.9%)
Ischemia acute/subacute	63 (1.4%)	0 (0.0%)	9 (0.5%)	54 (2.2%)
DAI	20 (0.4%)	4 (4.3%)	5 (0.3%)	11 (0.4%)
Tumor	61 (1.3%)	0 (0.0%)	8 (0.4%)	53 (2.1%)
Hydrocephalus	118 (2.6%)	0 (0.0%)	38 (1.9%)	80 (3.2%)
Extracranial	31 (0.7%)	0 (0.0%)	16 (0.8%)	15 (0.6%)
Other (cranial)	159 (3.5%)	1 (1.1%)	66 (3.4%)	92 (3.7%)

Percentage of all craniofacial abnormalities *SDH *subdural hematoma, *EDH *epidural hematoma, *SAH *subarachnoid hemorrhage, *DAI *diffuse axonal injury. Intraparenchymal hemorrhage = intracerebral hematoma + hemorrhagic contusion; Hydrocephalus = *CSF*-flow obstruction; Extracranial and Other = incidental extracranial and other cranial findings

Subdural hematoma, intraparenchymal hemorrhage, traumatic subarachnoid hemorrhage, midline shift, acute/subacute ischemia, and incidental intracranial tumors were more common in patients aged ≥ 65 years (generally 2–10 times higher incidence) compared with younger age groups. Conversely, epidural hematoma, skull fracture, facial fracture, pneumocephalus, and diffuse axonal injury were relatively more frequent in children and working-age adults.

### Co-occurrence of head and cervical spine injury

Concurrent acute abnormalities on both the head CT and the cervical spine CT were present in 126 of the 9,359 patients (1.3%). Isolated cranial or craniofacial abnormality occurred in 2,114 patients (22.6%), isolated cervical spine fracture in 349 patients (3.7%), and both examinations were negative for acute abnormalities in 6,770 patients (72.4%).

Overall, there was no statistically significant association between the presence of acute abnormalities on head CT and cervical spine fracture (χ² = 1.85, df = 1, *p* = 0.174). The odds ratio was 1.16 (95% confidence interval 0.94–1.43). However, the rate of concurrent injury increased significantly with age: 1/407 children (0.2%), 44/4,117 adults aged 18–64 years (1.1%), and 81/4,835 patients aged ≥ 65 years (1.7%) (*p* for trend = 0.002). Subgroup analysis showed a significant association only in the 18–64-year group (χ² = 7.55, *p* = 0.006), but not in children or the older adults (Table 4). Examples of trauma findings are presented in Supplementary Figure S1.

**Table 4:** Co-occurrence of craniofacial and cervical spine abnormalities

	Cranial finding	Spinal finding		*p*-value
Total		Present	Absent	
	Present	126	2,114	0.174
	Absent	349	6,770	
0–17		Present	Absent	
	Present	1	54	0.95
	Absent	6	346	
18–64		Present	Absent	
	Present	44	896	0.006
	Absent	91	3,086	
65**+**		Present	Absent	
	Present	81	1,164	0.54
	Absent	252	3,338	

### Alcohol intoxication

Alcohol intoxication was explicitly documented in the clinical referral in 1,501 of the 9,359 patients (16.0%): 19/407 children (4.7%), 1,094/4,117 adults aged 18–64 years (26.6%), and 388/4,835 patients aged ≥ 65 years (8.0%).

Documented alcohol intoxication was significantly more common among patients with abnormal head CT findings (417/2,240, 18.6%) than among those with normal head CT (1,084/7,119, 15.2%; χ² = 14.53, *p* < 0.001). This association was observed across all age groups.

In contrast, no significant association was found between documented alcohol intoxication and cervical spine fracture (13.1% in patients with fracture vs. 16.2% in patients without fracture; 60/457 vs. 1,441/8,902; χ² = 3.02, *p* = 0.082).

### Predictors of cervical spine fracture

A binary logistic regression model was developed to identify independent predictors of acute cervical spine fracture among patients undergoing concurrent head CT. Predictors were categorized by age (0–17, 18–64, ≥ 65 years), trauma mechanism (low: ground-level falls; high: falls from height, motor vehicle/pedestrian accidents; sports/active: bicycle, scooter, equestrian, sports), and cranial injury (intracranial bleeding, fracture, or no acute trauma). The model demonstrated moderate discrimination (area under the receiver operating characteristic curve = 0.67). Independent predictors included age ≥ 65 years (aOR 6.64, 95% CI 3.28–15.90, *p* < 0.001), high-energy mechanism (aOR 2.24, 95% CI 1.74–2.86, *p* < 0.001), and bony cranial injury (aOR 1.60, 95% CI 1.09–2.26, *p* = 0.011). Intracranial bleeding was not independently associated with cervical spine fracture.

In a second model using any acute traumatic finding (cranial or spinal) as the dependent variable, ground-level falls were associated with lower odds of acute traumatic findings (aOR 0.83, 95% CI 0.74–0.92, *p* < 0.001). In the fracture model, ground-level falls were similarly associated with lower odds of cervical spine fracture (aOR 0.76, 95% CI 0.61–0.94, *p* = 0.009).

### Management of cervical spine fractures

Of the 457 patients with acute cervical spine fractures, 366 (80.1%) were managed conservatively, and 91 (19.9%) underwent surgical stabilization during the index admission or subsequent follow-up. Thus, the overall rate of surgically treated cervical spine fractures was 1.0% (1.1% in those with abnormal head CT and 0.9% in those with normal head CT) among all scanned patients. Surgical treatment was more common in younger patients: 2/7 pediatric cases (28.6%), 32/133 adults aged 18–64 years (24.1%), and 57/317 patients aged ≥ 65 years (18.0%). Among 5,313 patients imaged after ground-level falls, 72 (1.4%) underwent surgical stabilization for cervical spine fracture: 0/38 (0%) pediatric patients, 9/1,448 (0.6%) adults aged 18–64 years, and 63/3,827 (1.7%) patients aged ≥ 65 years. Follow-up imaging (CT, MRI, or radiography) was performed in 324/457 (70.9%) patients with cervical spine fractures, including 7/7 pediatric patients, 95/133 adults aged 18–64 years, and 222/317 patients aged ≥ 65 years.

### Trends in imaging utilization, diagnostic yield, and trauma mechanisms

**Fig. 2:**
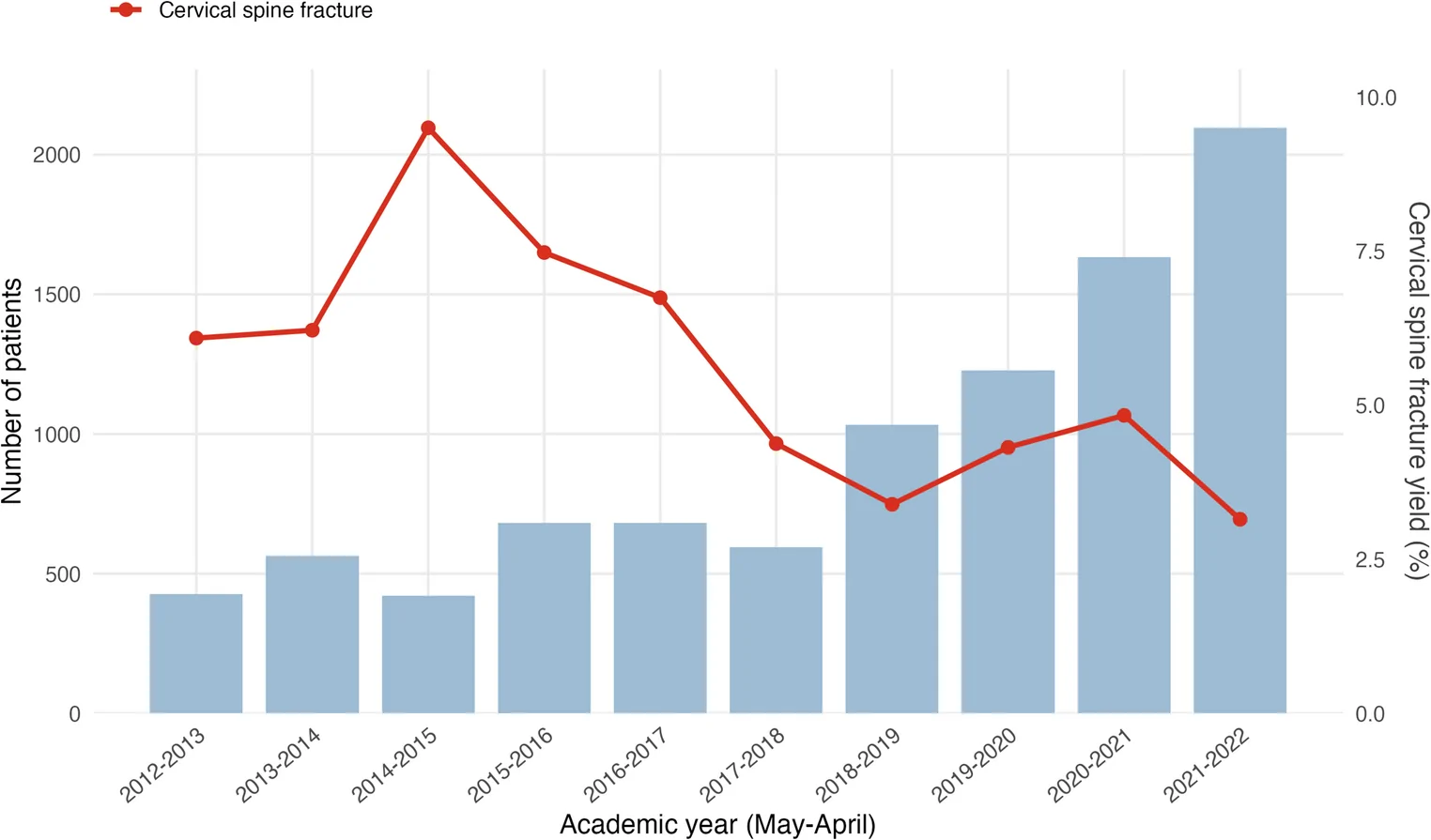
Annual number of patients undergoing concurrent head and cervical-spine CT examinations and corresponding cervical-spine fracture yield during the study period

The annual number of concurrent head and cervical spine CT examinations increased nearly fivefold over the study period, from 427 in the first year to 2,096 in the last year. In contrast, the annual number of acute cervical spine fractures remained relatively stable (range 26–79 per year), so the fracture yield declined from 7.1% in 2012–2017 to 3.9% in 2017–2022 (Fig. 2). Cranial or craniofacial abnormality yield declined from 28.5% to 22.0% over the same periods.

The decline in yields coincided with a marked increase in the proportion of examinations performed for ground-level falls (from 47% in the first year to 65% in the last). Among the 5,313 ground-level fall cases, acute cervical spine fractures were detected in 282 patients (5.3%) and acute cranial or craniofacial abnormalities in 1,295 patients (24.4%). Notably, the diagnostic yield within the ground-level fall cohort itself also declined significantly over the study period. Cervical spine fracture yield in ground-level falls decreased from 7.2–9.5% in the first five years to 3.6–5.3% in the last five years (annual change: − 0.59% per year, *p* = 0.0004). Similarly, cranial or craniofacial abnormality yield within this group decreased from 31.1–37.3% to 19.6–26.9% over the same periods (annual change − 1.76% per year, *p* = 0.0008).

Nearly all positive findings in ground-level falls occurred in adults and older patients. Pediatric cases yielded no cervical spine fractures and only four cranial abnormalities across the decade.

## Discussion

This retrospective analysis of 9,359 concurrent non-contrast head and cervical-spine CT examinations over 10 years revealed a marked increase in imaging volume accompanied by declining diagnostic yields. Acute cervical spine fractures were identified in 4.9% of examinations and intracranial or craniofacial abnormalities in 23.9%, while concurrent abnormalities on both examinations occurred in only 1.3%. There was no significant overall association between positive head CT findings and cervical-spine fracture (OR 1.16, *p* = 0.174). The decline in yield was particularly pronounced among patients presenting after ground-level falls, whose proportion increased from 47% to 65% of examinations over the study period. Within this low-energy mechanism, cervical fracture yield fell from 7–9% to 3–5%, and cranial yield from 31–37% to 20–27%. Nearly all positive findings occurred in adults and older patients, with pediatric ground-level falls yielding almost no abnormalities.

These patterns are consistent with broader international trends of increasing emergency department CT utilization, particularly for low-risk mechanisms in aging populations. Ground-level falls are now the leading cause of ED visits in older adults, and liberal imaging thresholds, often driven by defensive medicine, fear of missing occult injury, or protocol inertia, have led to substantial over-imaging without proportional detection of clinically important findings. Concurrent injury in both regions was rare in our cohort (1.3%), and head and cervical spine findings were statistically independent (OR 1.16, *p* = 0.174). An indication for imaging one region therefore does not in itself justify scanning the other.

Our findings therefore suggest that routine concurrent scanning may not be justified in most patients. However, we do not suggest that imaging should be withheld from older trauma patients. Rather, our findings highlight the limitations of current decision rules (particularly the Canadian C-Spine Rule, which mandates cervical imaging for patients aged ≥ 65 years) and the need for better risk stratification specifically in older patients with low-energy falls.

Multivariate analysis reinforced the limited utility of routine concurrent cervical-spine imaging. A logistic regression model incorporating age, mechanism energy level, and cranial injury severity demonstrated moderate predictive performance (AUC 0.67), suggesting limited discriminatory ability for identifying patients with cervical fracture. High-energy mechanisms and advanced age were the strongest predictors, whereas bony cranial injury showed only a modest association.

Notably, intracranial bleeds were not significant predictors. This contrasts with previously published similar data [15], which showed that acute intracranial lesions were associated with a higher risk of cervical fractures (OR 1.69, *p* = 0.04). This discrepancy may reflect changes in imaging thresholds, methodological differences, and case mix over time, particularly the rising proportion of low-energy ground-level falls, which dilute the predictive value of head findings for concurrent spine injury. Logistic regression analysis confirmed that ground-level falls (the fastest-growing subgroup in our study) were associated with significantly lower odds of CT-positive injury compared with higher-energy mechanisms, even after adjustment for age. The shift of ground-level falls from a minority to nearly two-thirds of total imaging volume largely explains the observed inverse relationship between scan volume and diagnostic yield. These results support targeted, rather than routine, cervical spine imaging guided by validated decision rules to reduce overutilization without compromising the detection of unstable injuries.

Management of the 457 cervical-spine fractures was predominantly conservative (80.1%), with surgical stabilization performed in 19.9% of cases and less frequently in the older adults (18.0%). In the ground-level fall subgroup, the overall surgical yield was low (1.4%), with age-stratified rates of 0% in children, 0.6% in working-age adults, and 1.7% in the older patients. Together with the observed decline in diagnostic yield, this indicates that the fivefold increase in imaging volume largely identified either no injury or stable fractures that were managed non-operatively. This aligns with current evidence favoring conservative management for many odontoid and stable subaxial fractures in older patients, where comorbidities often outweigh surgical risks. The high rate of follow-up imaging (70.9%) further reflects the resource burden of monitoring low-grade findings. Notably, ground-level falls in children resulted in zero cervical-spine fractures and zero surgical interventions, highlighting strong potential for imaging reduction in this group using established decision rules.

Documented alcohol intoxication was present in 16.0% of referrals and was significantly associated with abnormal head CT findings (18.6% vs. 15.2%, *p* < 0.001), consistent with the known increased risk of traumatic brain injury in intoxicated patients. No significant association was found with cervical spine fractures (*p* = 0.082), although the trend toward lower prevalence in fracture cases may reflect under-documentation or different injury mechanisms.

These findings align with international reports on paired head and cervical spine CT. Graterol et al. (2018) [5] found that nearly 90% of cervical-spine CTs in 2,888 blunt trauma patients were performed concurrently with head CT, yet clinically significant injury in both regions was detected in only 0.5%. Culhane et al. (2022) [16] reported a 9.9% overall cervical-spine injury rate among patients with ground-level falls (*n* = 3,520), but CT alone missed 0.3% of unstable injuries requiring surgery (negative predictive value of 99.7%). A recent systematic review and meta-analysis by McCallum et al. (2023) [17] found a pooled prevalence of any cervical spine injury of 3.8% (95% CI 2.8–5.3) in adults aged ≥ 65 years after low-level falls. Although these prevalences are not negligible and indicate that imaging of appropriately selected older patients with fall history may have real clinical value, the studies are broadly consistent with the relatively low yields observed in our ground-level fall subgroup. Earlier work by Griffith et al. (2011) [18] and Baker et al. (2020) [19] similarly documented low yields (2–5%) for cervical spine injury in emergency department screening and highlighted frequent over-utilization, especially in low-risk or low-energy presentations.

Taken together, these findings suggest that while imaging remains important in selected patients, the diagnostic value of routine concurrent cervical-spine imaging may be limited in low-energy trauma. Greater use of validated clinical decision rules, including NEXUS and the Canadian C-Spine Rule, may help reduce unnecessary imaging, although their effectiveness specifically in patients undergoing concurrent head and cervical-spine CT remains uncertain.

Strengths of this study include its large, consecutive cohort of 9,359 patients spanning a full decade, enabling assessment of temporal trends and age-specific patterns. Multivariable logistic regression identified independent predictors of cervical-spine fracture while accounting for key factors such as age and trauma mechanism. The study uses real-world emergency department data with a standardized dual-read protocol by subspecialized neuroradiologists, supporting high diagnostic reliability. Detailed manual extraction of trauma mechanisms and management decisions provides a direct link between imaging findings and clinical consequences.

Several limitations should be acknowledged. As a single-center retrospective study, the results reflect local imaging thresholds and trauma profiles that may not fully generalize to other settings. Reliance on clinical referral texts for trauma mechanism and alcohol status likely underestimates the true prevalence of intoxication, as documentation was not systematic. Furthermore, the brevity and variable detail in referrals mean that specific clinical reasoning (including use of validated decision rules such as NEXUS or the Canadian C-Spine Rule) remains unknown. Consequently, the relative contribution of protocol-based practice versus individual clinical judgment to the five-fold increase in imaging volume cannot be determined. Imaging findings were based on original clinical reports rather than blinded re-review, although the institutional second-read protocol by subspecialized neuroradiologists helps mitigate observer bias. Most examinations used standard head and cervical-spine protocols without dedicated facial bone imaging, potentially underestimating facial fracture incidence. In addition, only osseous injuries were systematically recorded; purely ligamentous or distractive cervical injuries without fracture, such as disc-space or facet-joint widening that may indicate instability, were not separately coded and may therefore have been underrepresented. Finally, while immediate management decisions (surgical vs. conservative) were captured, the lack of long-term clinical outcomes or data on missed injuries limits conclusions regarding chronic morbidity.

In conclusion, the marked increase in concurrent head and cervical spine CT utilization, driven by low-energy falls in an aging population, has been accompanied by declining diagnostic yield and limited added value from routine dual imaging. These findings support greater adherence to validated clinical decision rules and more selective imaging strategies to reduce unnecessary radiation exposure, healthcare costs, and emergency department resource utilization while maintaining patient safety. Future prospective studies evaluating rule-based protocols in similar high-volume settings would be valuable.

## Supplementary Information

Below is the link to the electronic supplementary material.

Supplementary Material 1

## Data Availability

The datasets generated and analysed during the current study are not publicly available due to institutional policies and national regulations protecting patient privacy and confidentiality. De-identified data may be made available from the corresponding author upon reasonable request and subject to approval by the institutional review board of Tampere University Hospital.
